# Clinical outcome and prognostic factors of patients with early-stage epithelial ovarian cancer

**DOI:** 10.18632/oncotarget.13317

**Published:** 2016-11-11

**Authors:** Wei Wei, Ning Li, Yangchun Sun, Bin Li, Lily Xu, Lingying Wu

**Affiliations:** ^1^ Department of Gynecologic Oncology, National Cancer Center/Cancer Hospital, Chinese Academy of Medical Sciences and Peking Union Medical College, Beijing, China; ^2^ Chemistry Department, Wellesley College, Wellesley, MA, USA

**Keywords:** ovarian cancer, early-stage, prognosis, survival, recurrence

## Abstract

Some subsets of early stage ovarian cancer patients experience more recurrences than others. Studies on prognostics factors gave conflicting results. We investigated consecutive 221 patients with stage I/II ovarian cancer at our institution from 1999 to 2010. Univariate and multivariate analysis of progression free survival (PFS) and overall survival (OS) were performed. After a median follow-up of 79 months, the 5-year/10-year PFS and 5-year/10-year OS were 78% /76% and 90% /87% respectively. Multivariate analysis revealed that stage as the most prominent independent prognostic factor in terms of PFS (stage I *vs* stage IIA *vs* stage IIB, Hazard Ratio (HR): 1 *vs* 4 *vs* 6.1, *P* < 0.05) and OS (stage I *vs* stage II, HR: 1 *vs* 2.1, *P* < 0.05). Peritoneal biopsy reduced the risk of recurrence by 29% (95% CI: 0.15-0.58, *P* < 0.05). Ascites (HR = 2.8, 95% CI: 1.2-6.6, *P* < 0.05) and not the first-line chemotherapy (HR = 2.6, 95% CI: 1.1-6.5, *P* < 0.05) contributed to decreased OS. Overall, early-stage ovarian cancer had a favorable outcome, stage was the most powerful prognostic factor.

## INTRODUCTION

Ovarian cancer is the second most common gynecological cancer worldwide [1]. In China, it is the leading cause of death from gynecologic cancer with approximately 52,100 new cases and 22,500 deaths in 2015 and with an estimated incidence to mortality rate of 43.1% [2]. Ovarian malignancies are surgically staged according to the International Federation of Gynecology and Obstetrics (FIGO) staging criteria [3]. The survival rate declines dramatically when the disease spreads out of the pelvic cavity and develops into advanced stages (FIGO stages III and IV) [3]. The reported 5-year survival rate is approximately 10%-30 % for advanced stages and 85%-90% for early stage (FIGO stage I-II) [4]. Unfortunately, more than 70% of patients are diagnosed with advanced stages [5].

Although the overall outcomes for early-stage EOC is generally optimistic, the actual reported recurrence rate ranges from 10% to 50% [6]. Clearly, some subgroups of early-stage EOC with unfavorable prognostic factors experience more relapses. Consequently, postoperative chemotherapy is required in high risk early stage patients. The major high risk factors for early stage patients include stage IC or higher, clear cell type, and poor differentiation according to current clinical practice guideline [7]. In contrast, fertility sparing surgery (FSS) is proved to be safe for selected patients [8]. Potential clinical and pathological factors are still being explored to tailor the treatment of early stage EOC patients. Recently, a model divided epithelial ovarian cancer into type I and type II ovarian cancer has been introduced. Type I tumors are slow growing, generally stage I at diagnosis and developed from well established precursor lesions [9]. FIGO staging criteria has been updated in 2014, emphasizing the different patterns of tumor rupture and positive cytology [10]. In this study, we restaged the EOC patients with the long-term follow-up using the latest staging system and took into account some new perspectives, aiming to help optimize the treatment for early stage EOC.

## RESULTS

**Figure 1:**
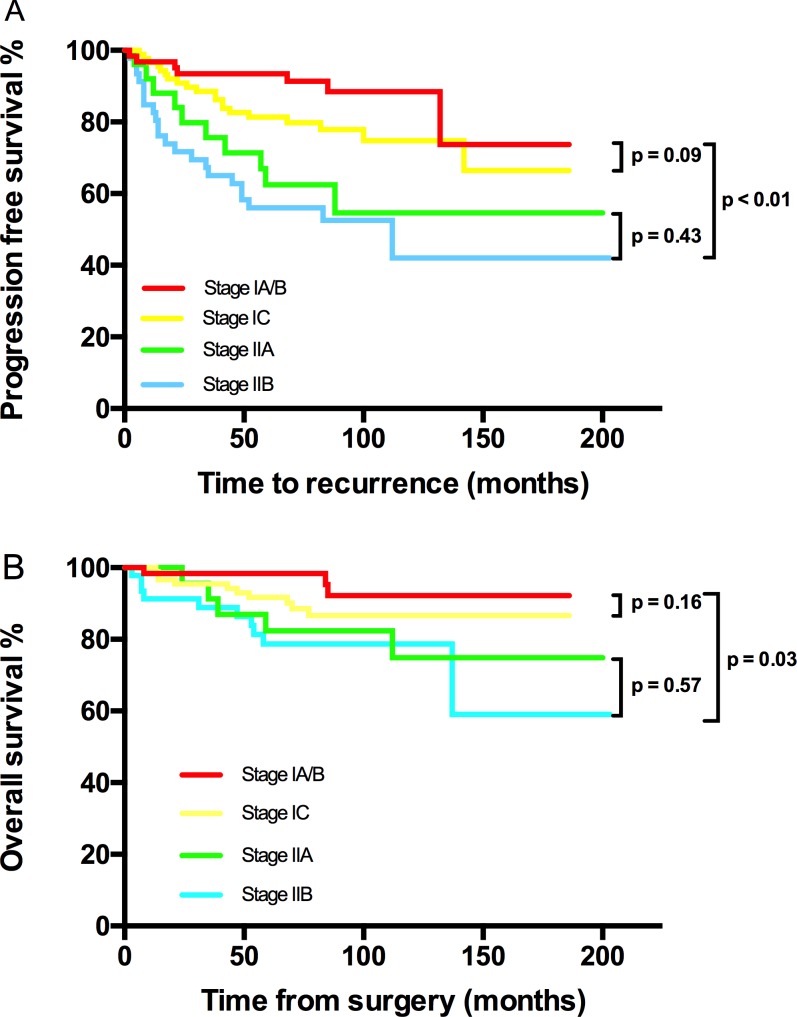
Kaplan-Meier estimated PFS and OS stratified by FIGO stage **A**. Progression free survival calculated by log-rank test. (stage IA/B *vs* stage IC vs stage IIA vs stage IIB, *P* < 0.01; stage IA/B vs stage IC, *P* = 0.091; stage IIA vs stage IIB, *P* = 0.43) and **B**. Overall survival calculated by log-rank test. (stage IA/B vs stage IC vs stage IIA vs stage IIB, *P* < 0.01; stage IA/B vs stage IC, *P* = 0.16; stage IIA vs stage IIB, *P* = 0.572).

221 patients were included in this retrospective analysis. The median duration of follow-up was 79 months. 59 individuals experienced recurrence and 28 were reported as demised. The 5-year PFS and 10-year PFS were 78% (95% CI: 73-84) and 76% (95% CI: 70-82) respectively (Figure 1). For relapsed patients, the median PFS time was 24 months, ranging from 2 months to 142 months. The overall 5-year and 10-year OS were 90% (95% CI: 86-94) and 87% (95% CI: 83-91) respectively.

The median age at diagnosis was 47 years. Table 1 lists the main clinical and pathologic characteristics. In general, 151 (68.3%) patients had stage I disease, with stage IA in 59 (26.7%), IB in 4 (1.8%), and IC in 88 (39.8%). 70 (31.6%) patients developed stage II cancers, with IIA in 25 (11.3%) and IIB in 45 (20.3%). Additionally, stage IC group were classified as IC1 (23/21.6%), IC2 (41/46.5%) and IC3 (24/31.8%). The most common histologic type was serious adenocarcinoma (40.3%), followed by mucinous adenocarcinoma (19.9%), clear cell cancers (15.4%), endometrioid cancers (16.7%) and other types (7.7%). The frequency distributions of the differentiation were well-differentiation (G1) (34.4%), moderate-to-well differentiation (G2-3) (50.2%), and clear-cell type, which was considered a specific differentiation group, accounting for 15.4%. One hundred and twenty-two (55%) had penetrated or ruptured tumor capsules and 59 (26.7%) had positive ascites or washing cytology. Nine (4.1%) patients reported a positive family history of ovarian cancer and/or breast cancer and 35 (15.8 %) patients were diagnosed with other types of cancers during the follow-up. The majority (60.6%) of participants did not present obvious ascites.

**Table 1:** PFS and OS univariate analysis by patients’ characteristics

		N(%)	5/10-year PFS	P	5/10-year OS	P
**Age (years)**	≤ 30	26(11.8)	96%/96%	**0.03**	100%/100%	0.12
	30-60	159(22.6)	75%/68%		88%/82%	
	≥60	36(65.6)	74%/70%		88%/82%	
**Ascites**	No	134(60.6)	83%/76%	**0.02**	94%/91%	**<0.01**
	Yes	87(39.4)	68%/63%		83%/71%	
**FIGO Stage**	IA/B	63(28.5)	94%/88%	**<0.01**	92%92/%	**0.03**
	IC	88(39.8)	81%/75%		92%/87%	
	IIA	25(11.3)	63%/55%		82%/75%	
	IIB	45(20.3)	56%/42%		79%/79%	
**Capsule rupture**	No	99(44.8)	86%/76%	0.08	96%/90%	0.05
	Yes	122(55.2)	72%/67%		85%/80%	
**Cytology**	Negative	150(67.9)	86%/78%	**<0.01**	94%/87%	0.09
	Positive	59(26.7)	60%/52%		83%/80%	
	Unknown	12(5.4)	58%/58%		73%/73%	
**Type^a^**	I	112(50.7)	84%/76%	**0.04**	90%/87%	0.07
	II	97(43.9)	71%/64%		90%/81%	
	Undefined	12(5.4)	62%/62%		91%/91%	
**Histological type**	Serous	89(40.3)	72%/64%	0.07	90%/86%	0.05
	Mucinous	44(19.9)	91%/91%		98%/97%	
	Clear cell	34(15.4)	68%/62%		82%/77%	
	Endometrioid	37(16.7)	86%/71%		88%/73%	
	Other	17(7.7)	69%/69%		87%/79%	
**Differentiation**	Good	76(34.4)	89%/82%	**0.01**	95%/92%	0.08
	Moderate-Poor	111(50.2)	72%/65%		90%/82%	
	Clear cell	34(15.4)	68%/61%		82%/77%	
**FSS^b^**	No	202(91.4)	75%/68%	**0.01**	90%/83%	0.09
	Yes	19(8.6)	100%/100%		100%/100%	
**Peritoneal biopsy**	No	41(18.5)	66%/54%	0.06	84%/80%	0.27
	Yes	120(81.5)	79%/75%		94%87/%	
**Lymphadenectomy**	Undone	72(32.6)	80%/74%	0.94	91%/89%	0.50
	Systematic	129(58.4)	76%/67%		89%/81%	
	Unsystematic	20(9.0)	75%/66%		89%/84%	
**Residual mass**	No	201(91.0)	80%/76%	**<0.01**	92%/87%	**<0.01**
	Yes	20(9)	50%/50%		69%/58%	
**Chemotherapy^c^**	No	25(11.3)	92%/82%	**0.01**	89%/89%	**0.01**
	PTX+CBP/DDP	168(76.0)	77%/73%		91%/87%	
	CTX+CBP/DDP	20(9.0)	75%/68%		80%/72%	
	Other	8(3.6)	47%/23%		60%/60%	
**Courses of Chemotherapy**	0	25(11.3)	82%/82%	0.29	90%/90%	0.35
	1-3	27(12.2)	77%/72%		85%/78%	
	>3	169(76.5)	75%/68%		89%/84%	

a: Type: low-grade serous tumor, low-grade endometrioid tumor, clear cell tumor and Type II includes mucinous tumor are Type I; its high-grade tumors are type II. b: FSS: Fertility sparing surgery. c: PTX: Paclitaxel, CBP: Carboplatin, DDP: Cisplatin, CTX: cyclophosphamide.

All patients underwent total hysterectomy, bilateral-oophorectomy and omentectomy unless FSS was required. 81.5% patients received peritoneal biopsy and 68% had pelvic lymph node dissection (sampling or systematical removal). We analyzed factors that might influence the completion of peritoneal biopsy and lymphadenectomy. Stage and tumor capsule penetration/rupture did not reveal correlation; nevertheless, greater age and positive peritoneal cytology increased the rate of pelvic lymphadenectomy (*P* = 0.03 and *P* = 0.01 respectively). Optimal cytoreductive surgery (residual mass < 1cm) was achieved in 97.5% patients. Furthermore, 201 (91.0%) patients were left with macro-invisible implants after the dissection. Notably, 15 stage IA and 2 stage IC3 patients underwent FSS; and 11 of them received at least 3 cycles of adjuvant chemotherapy. After a minimal 59-month follow-up, no recurrence was observed amongst FSS patients. 89% patients were administrated adjuvant chemotherapy. Among all 25 patients (23 stage IA and 2 stage 1C) exempted from chemotherapy, no clear cell type and grade 3 tumor was identified.

**Figure 2:**
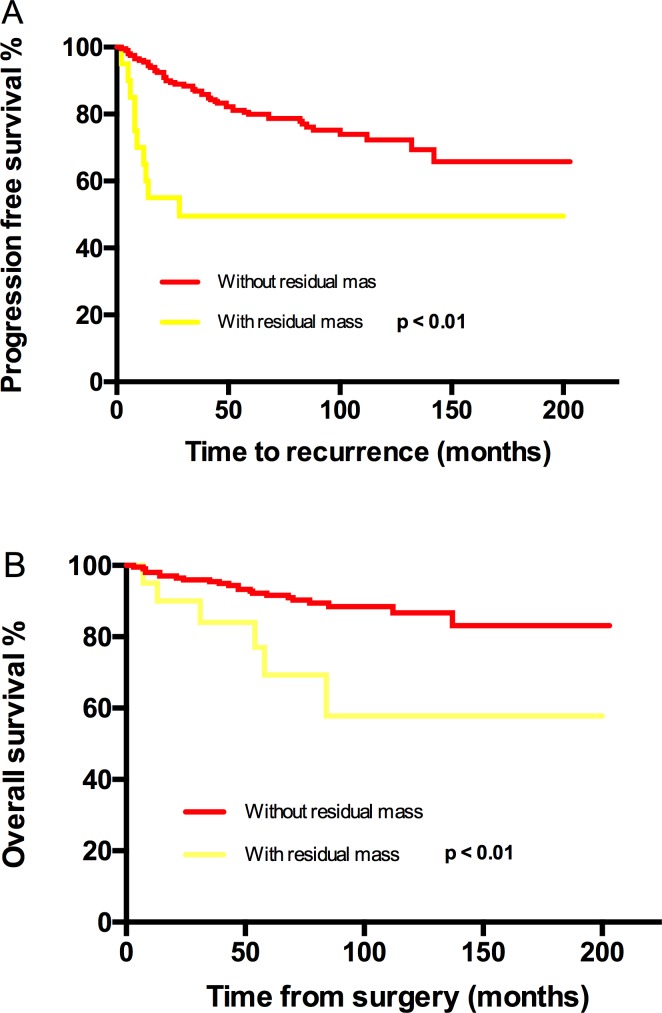
Kaplan-Meier estimated PFS and OS stratified by residual mass **A**. Progression free survival calculated by log-rank test. (without residual mass vs with residual mass, *P* < 0.01) and **B**. Overall survival calculated by log-rank test. (without residual mass vs with residual mass, *P* < 0.01).

**Figure 3:**
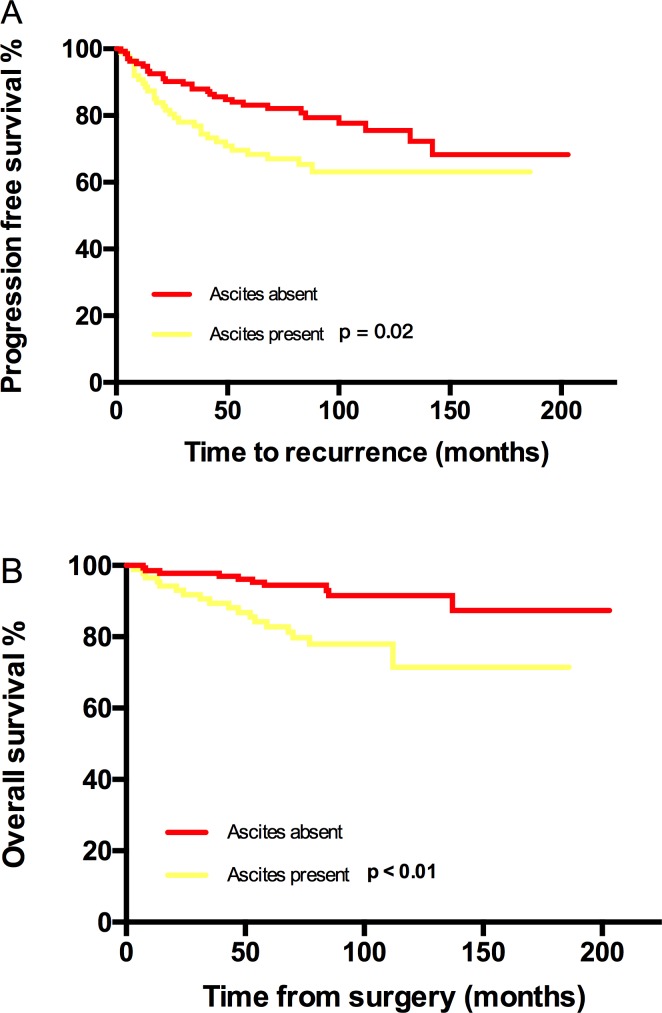
Kaplan-Meier estimated PFS and OS stratified by ascites **A**. Progression free survival calculated by log-rank test. (ascites absent vs ascites present, *P* = 0.02) and **B**. Overall survival calculated by log-rank test. (without residual mass vs with residual mass, *P* < 0.01).

PFS and OS differences among stage IC subgroups were not detected, thus stage IC was analyzed as an entity. An earlier stage, no visible residual mass, and no ascites were associated with both increased PFS and OS. The 5-year/10-year PFS from stage IA/B, IC, IIA to IIB were 94%/88%, 81%/75%, 63%/55% and 56%/42% (*P* < 0.01) respectively; the corresponding 5-year/10-year OS were 92%/92%, 92%/87%, 82%/75% and 79%/79% (*P* = 0.03) (Figure 1). However, the significant differences of PFS and OS were not detected when compared stage IA/B and stage IC or stage IIA and stage IIB (Figure 1). Visible residual mass decreased 5-year/10-year PFS (50%/50% *vs* 80%/76%, *P* < 0.01) and 5-year/10-year OS (92%/87% *vs* 69%/58%, *P* < 0.01) (Figure 2). Ascites was also an unfavorable prognostic factor; the statistics were 83%/76% *vs* 68%/63% for 5-year/10-year PFS (*P* = 0.02) and 94%/91% *vs* 83%/71% for 5-year/10-year OS (*P* < 0.01) (Figure 3). Additionally, younger age (*P* = 0.03), negative cytology (*P* < 0.01), type I (*P* = 0.04), and good differentiation (*P* = 0.01) all improved 5-year/10-year PFS but not OS. Patients received FSS were associated with prolonged PFS, but it reflected mostly the characteristics of patients rather than the surgical procedure. On the contrary, a ruptured tumor with marginal P value (*P* = 0.05) may only influence OS but not PFS. Histology had marginal P values for both PFS (*P* = 0.07) and OS (*P* = 0.05). There were significant differences regarding PFS (*P* = 0.01) and OS (*P* = 0.02) when we stratified tumors by mucinous type, indicating that mucinous histology was a favorable prognostic factor. The clear cell histology failed to be associated with significantly worse PFS and OS, but a trend toward inferior outcomes was found.

On multivariate analysis, we included prognostic factors with significant or marginal P values. However, age and FSS were omitted from the model because they were closely related with the stage. Histologic type and differentiation were incorporated instead of type I and II ovarian cancer. An earlier stage and peritoneal biopsy independently decreased the risk of recurrence. Compared with stage IA/B, stage IC had a similar risk of recurrence. On the other hand, stage IIA suffered a 4 times greater (95% CI:1.2-13.3, *P* < 0.01) risk of recurrence than stage I, and the risk increased to 6.1 times (95% CI:1.9-19.1, *P* = 0.03) for stage IIB. Hazard ratio (HR) for peritoneal biopsy group was 0.29 (95% CI: 0.15-0.58, *P* < 0.01). More factors that independently impacted OS were found. The differences between stage IA/B and stage IC, stage IIA and IIB were not statistically obvious. Nevertheless, stage II elevated the risk of death by 2.1 times (95% CI: 2.1-3.5, *P* = 0.08). Peritoneal biopsy reduced the risk of death by 67% (95% CI: 0.11-0.96, *P* = 0.04). Compared with the serous group, the clear cell type (HR = 6.0, 95% CI: 1.7-22, *P* = 0.01) and endometrioid histology (HR = 5.4, 95% CI: 1.7-16, *P* = 0.01) were associated with higher risk of death. Lastly, ascites (HR = 2.8, 95% CI: 1.2-6.6, *P* = 0.16) and not the first-line chemotherapy (HR = 2.6, 95% CI: 1.1-6.5, *P* = 0.03) were associated with increased risk of death. The results of multivariate analysis are listed in Table 2.

**Table 2:** PFS and OS multivariate analysis by patients’ characteristics

		Progression free survival				Overall survival		
		Hazard Ratio	95% CI^a^	P		Hazard Ratio	95% CI	P
**Ascites**	**No**	1.0		0.13		1.0		**0.02**
	**Yes**	1.6	0.86-2.7			2.8	1.2-6.6	
**Peritoneal Biopsy**	**No**	1.0		**<0.01**		1.0		**0.04**
	**Yes**	0.29	0.15-0.58			0.33	0.11-0.96	
**Residual mass**	**No**	1.0		0.29		1.0		0.07
	**Yes**	1.6	0.68-3.6			2.7	0.92-8.2	
**FIGO Stage**	**IA+IB**	1.0		**<0.01**		-	-	**-**
	**IC**	1.6	0.56-4.6	0.38		-	-	-
	**IIB**	6.1	1.9-19.1	**<0.01**		-	-	-
	**IIA**	4.0	1.2-13.3	**0.03**		-	-	-
	**I**	-	-	-		1.0		**0.01**
	**II**	-	-	-		2.1	1.2-3.5	
**Capsule rupture**	**No**	1.0		0.70		1.0		0.48
	**Yes**	0.88	0.45-1.7			1.4	0.56-3.4	
**Cytology**	**Negative** **/Unknown**	1.0		0.15		1.0		0.61
	**Positive**	1.5	0.86-2.7			1.3	0.54-2.9	
**Differentiation**	**G1^b^**	1		0.09		1		0.92
	**G2-3^c^/Clear cell**	1.4	0.95-2.1			1.0	0.58-1.8	
**Histologtic type**	**Serous**	1.0		0.59		1.0		**0.01**
	**Mucinous**	0.96	0.31-3.0	0.75		0.65	0.07-6.4	0.53
	**Clear cell**	1.96	0.86-4.1	0.54		6.0	1.7-22.0	**0.01**
	**Endometrioid**	1.31	0.56-3.1	0.11		5.4	1.7-16.0	**0.01**
	**others**	17	0.47-2.9	0.95		1.3	.33-5.5	0.68
**Chemotherapy**	**PTX+CBP/DDP^d^**	1.0		0.07		1.0		0.10
	**No**	1.5	0.41-5.7	0.53		1.7	0.19-14.7	.65
	**Other**	2.0	1.1-3.9	0.24		2.6	1.1-6.4	0.**03**

a: CI: Confident interval; b: G1, well differentiated; c:G2, moderately differentiated, G3, poorly differentiated; d: PTX: Paclitaxel, CBP: Carboplatin, DDP: Cisplatin.

## DISCUSSION

Study on prognosis of early stage EOC is sufficient because it is less common and requires longer follow-up period than advanced-stage counterpart. A limit number of researches has been carried since the FIGO staging system updated in 2014. A dualistic type I/II model of ovarian carcinogenesis has been proposed in recent years, but the prognostic role of this model in early-stage EOC is still unclear. With respects to these progresses, we investigated early stage EOC patients in our institution with long-term follow-ups.

In our study, tumor stage was the most prominent independent factor for PFS and OS. The 5-year/10-year PFS were 86.5%/80.7% for stage I and 57.7%/48.2% for stage II (P < 0.01). Correspondingly, a study including 114 patients who received primary surgery and first-line chemotherapy demonstrated 85% and 44% 5-year PFS for stage I and stage II EOC patients respectively [11]. Another similar large scale clinical trial reported PFS rates of 82% and 67% for women diagnosed with FIGO stage I and stage II disease respectively [12]. It is noticeable that women with a stage II disease had an obvious lower progression-free survival rate compared with stage I in most studies. Additionally, in our study, the 5-year/10-year OS were 94.5%/89.1% and 79.6%/74.9% for stage I and II respectively (*P* = 0.01). A study in which 457 early-stage EOC patients received at least 3 cycles chemo therapeutic agents after primary surgery, the calculated probability of survival was 5 years at 84% (stage I) and 73% (stage II) [12]. Another similar clinical study had 5 years OS of 88.8% and 78.9% for stage I and stage II respectively [13]. Several studies reported 5-year OS ranging from 79%-85% in stage I patients who received various adjuvant chemotherapy [14–16]. The discrepancies of reported overall survival rates contributed in part to different baseline characteristics and treatment for these studies. Still, compared with other studies, the overall survival rates in our study were optimistic. In our institution, maximum effort was exerted to remove all metastasis after recurrence by a flexible combination of chemotherapy and multi-disciplinary cytoreductive surgery. We assumed that these partially explain the generally favorable overall survival in our patients. We did not reveal statistical differences for both PFS and OS within stage I and within stage II; it could partly be due to the small event numbers in our study. The stage was revealed as an independent prognostic factor in our investigation. Contrarily, some authors indicated that stage was not influential if optimal treatments were administrated in early stage patients [17]. The latest FIGO staging criteria further classified stage IC, stressing the influences of surgical tumor spill (stage IC1), tumor capsule rupture before surgery or tumor on ovarian surface (stage IC2), and positive ascites or peritoneal washings (stage IC3). However, significant differences between 5-year/10-year PFS (IC1 *vs* IC2 *vs* IC3, 70%/70% *vs* 85%/78% *vs* 89%/81%, *P* = 0.46) and 5-year/10-year OS (IC1 *vs* IC2 *vs* IC3, 85%/78%, 98%/91%, 90%/90%, *P* = 0.34) among IC subgroups were not distinguished in our study. Similarly, a meta-analysis of suggested that stage IC had a similar outcome as stage IB disease [18]. Additionally, both tumor rupture and positive cytology showed significant or marginal P values on univariate analysis but not on multivariate analysis in our study, indicating that the effects of tumor rupture and positive cytology may be reduced if appropriate treatments exist. Similar conclusion was draw by a meta-analysis conducted by Kim et al [19].

Involvement of pelvic nodes have been reported to occur in 8-15% and of para-aortic nodes in 5-24% of patients with implants macro-optically confined to ovaries [20–22]. Although Lymphadenectomy could increase the accuracy of staging and reduce the rate of metastasis, it was not a standard procedure at the start. Lymphatic dissection did not improve the prognosis in our study, though, which was consistent with some other studies [23]. We assumed that this is primarily because patients in our study all had negative lymph nodes, thus the importance of lymph dissection was undervalued. A study suggested that benefit of lymphadenectomy in early-stage EOC was more prominent for tumors with poor differentiation [24]. On the contrary, peritoneal biopsy was shown to be an independent factor for both PFS and OS. It is rational to speculate that positive peritoneal biopsy upgrades some stage I patients to stage II. The improved accuracy of staging might increase overall survival by prompting postoperative chemotherapy [25]. Notably, 14 out of 19 FSS patients did not have completed staging surgery because the intraoperative frozen pathology reports were negative; 8 patients were not given chemotherapy. None of FSS patients were reported recurrent. On one hand, our study suggested the significance of completion of staging surgery; on the other hand, we supported that FSS with or without chemotherapy might be a safe option for selected patients.

In line with advanced EOC, residual mass was found to be related to recurrence and death by a univariate test in our study; the 5-year/10 year PFS and corresponding OS were dramatically reduced from 80%/76% to 50%/50% (*P* < 0.01) and from 92%/87% to 69%/58 (*P* < 0.01) respectively. 75% of patients with residual mass in current study were stage IIB. After adjusting for stage, residual mass was not a prognostic factor for recurrence (*P* = 0.50) and overall death (*P* = 0.34).

Clear cell ovarian cancer is regarded as a poorer subtype which requires adjuvant chemotherapy in recent clinical practic [26]. Our study did not reveal the distribution of histology has a prognostic effect. When we separately analyzed the four main histologic subtypes by univariate survival analysis, the prognosis of clear cell type was not significantly inferior to the non-clear cell type's; a trend towards poorer outcomes could be shown, though. Surprisingly, the mucinous type neoplasm showed a declined rate of recurrence and death, but, the prognostic advantage of mucinous type did not maintain in the cox regression. Further analysis suggested that the mucinous group is correlated with well-differentiation and earlier stage in our study. Therefore, the prognostic advantage of mucinous type is more likely the results from the differentiation and grade. Some previous studies also reported relatively good prognoses for early stage mucinous cancer [4, 27]. Type I ovarian cancers are mostly diagnosed at early stage and typically indolent. Our study supported the observation that the type I ovarian cancer has a better clinical course [28–29]. However, type I was not a significant factor in the cox-regression model.

The main limitation of this study is the heterogeneity among patients. Treatment during the 1999-2005 period in our institution has improved in terms of operation and chemotherapy, but, not all patients underwent completed staging surgery, thus some of them might be under-graded. 12 cased missed cytology exam and accurate staging for them became impossible. The paclitaxel plus platinum regiment was not commonly administrated before 2002. However, the outcomes of our study confirmed the good prognosis of early stage ovarian cancer, suggesting clinical importance of some controversial factors. Further prospective studies regarding these factors are required to tailor the treatment strategy for early stage EOC patients.

## PATIENTS AND METHODS

All charts of early stage EOC patients treated at Peking Union Medical College, Cancer Institute during the period 1999 to 2010 were reviewed. The statuses of patients were obtained by telephone follow-up and records of the out-patients department. This analysis had appropriate IRB approval. Patients were surgically and pathologically staged by the FIGO staging system (2014). Patients with ovarian borderline tumors, concurrent or previous malignant disease, or disease of stage II or higher were excluded. All patients were untreated before surgery.

The main data collected in this study included patient characteristics at the diagnosis, detailed surgical and pathological report and postoperative treatment. Peritoneal cytology exams were performed using ascites or peritoneal washing. The role of systematic pelvic and para-aortic lymphadenectomy was not well established for early stage EOC during the studied time. Para-aortic nodes were not routinely dissected in our institution before 2005 and were sampled only when suspected lymphatic metastasis was presented. Systematic pelvic lymphadenectomy refers to a bilateral removal of lymph nodes around common iliac vessels, external iliac vessels, internal iliac vessels, and obturator fossa. For selected stage I patients, fertility preservation surgeries were adopted. Postoperative chemotherapy was administrated depending on high-risk factors and patient preference. Only after 2002 was platinum-based (cisplatin or carboplatin) chemotherapy used as first-line medication in our institution. Type I EOC is composed of low-grade serous tumor, low-grade endometrioid tumor, clear cell tumor, and mucinous tumor; its high-grade tumors are type II [28]. Recurrence was diagnosed by positive imaging examination with or without elevated CA-125; it was further confirmed by either exploratory surgery or chemotherapy reaction.

Overall survival (OS) was interpreted as the date from surgery to the time of death from any cause or the date of the last follow-up. Progression free survival (PFS) was calculated as the time from initial treatment to the first sign of relapse or the date of death. T tests and chi-square test were performed to compare values and proportions between groups respectively. Survival curves were depicted using the Kaplan Meier method. The survival functions of different factors were analyzed with the log-rank test and Cox regression models. *P* < 0.05 for a bilateral test was considered statistically significant. Analysis was performed using the SPSS 20.0 software.
